# Re-engaging students in medical school accreditation: the independent student analysis action (ISA2) approach

**DOI:** 10.15694/mep.2020.000015.1

**Published:** 2020-01-15

**Authors:** Kirstin W. Scott, Adam G. Berger, Jessica C. Stuart, Edward M. Hundert

**Affiliations:** 1 Harvard Medical School; 2 Brigham & Women's Hospital

**Keywords:** undergraduate medical education, accreditation, United States, Liaison Committee on Medical Education, student engagement, continuous quality improvement

## Abstract

This article was migrated. The article was marked as recommended.

**Background:** Student satisfaction with key aspects of a medical school program plays a major role in an institution’s accreditation in the United States (US). There is limited evidence regarding how to best engage students in responding to areas of lower student satisfaction that are identified through the national Liaison Committee on Medication Education (LCME) accreditation self-study process.

**Methods:**We present a student-led innovation to promote greater levels of student engagement throughout Harvard Medical School’s re-accreditation experience, which we refer to as the Independent Student Analysis Action (ISA ^2^) process. This innovation built directly upon the Independent Student Analysis (ISA) survey, which is expected by the LCME for accreditation. The ISA ^2^ process allowed medical student leaders to leverage ISA results to identify 11 priority areas that had relatively lower levels of satisfaction and subsequently coordinate focused, time-limited ISA ^2^ working groups to address these problematic areas. These working groups then presented their solutions to the student body, and a follow-up survey gauged satisfaction with these areas in light of the changes made.

**Results:** The ISA ^2^ process engaged over 110 students, faculty, and staff. The majority of the student body completed the follow-up survey, which demonstrated higher levels of satisfaction with these previously problematic areas as identified in the original ISA survey. Further, 96% of students reported being satisfied with the ISA ^2^ process as a mechanism for utilizing student feedback in the ISA to create meaningful institutional changes.

**Conclusions:** The ISA ^2^ served as a powerful convening mechanism for engaging a large number of students in our institution’s re-accreditation efforts. Other medical schools looking to involve students in their continuous quality improvement systems and accreditation experience may benefit from reviewing and customizing this model to their institution’s needs.

## Background

How medical students perceive their medical training plays a major role in the evaluation of medical school programs in the United States (U.S.). The Liaison Committee on Medical Education (LCME) is the entity responsible for accrediting U.S. medical school programs. A required component of the extensive re-accreditation process is a student-led survey, called the independent student analysis (ISA), which captures student input on key institutional domains that are relevant to the twelve LCME standards (e.g., academic and learning environment; curricular content) ( Liaison Committee on Medical Education, 2019). The ISA is designed to inform each institution’s approximately year-long “self-study” process that takes place prior to the official LCME site visit. During the site visit, external evaluators come to the school to meet with institutional leadership, students, and staff and compose a final recommendation to the LCME regarding whether or not the institution’s medical program is satisfactorily meeting accreditation standards.

The LCME places particular emphasis on achieving a high response rate to the ISA, which is reviewed at multiple stages, including during the site visit and when the LCME votes on the school’s accreditation determination. This is important since other sources of institutional student input tend to be limited by lower response rates and stem from a more restricted survey population (e.g., one class year versus the entire student body), such as the Association of American Medical Colleges (AAMC) Graduate Questionnaire (GQ) ( Association of American Medical Colleges (AAMC), 2019). Though the response rate for each institution varies, in 2019, approximately 84% of all graduating U.S. medical students completed the AAMC GQ. Another notable difference is that while both the AAMC GQ and ISA contain standardized, required questions, the student authors of each institution’s ISA are free to develop any additional questions they believe are relevant to their unique curriculum and school culture ( Liaison Committee on Medical Education, 2019). Further, compared to the AAMC GQ, the ISA survey captures real-time feedback across the entire, current student body that is ripe for intervention during the school’s LCME re-accreditation self-study process.

Though the traditional ISA survey has many strengths for capturing student voices in the accreditation process, it has some important limitations. First, while the ISA survey is led entirely by students, their role typically ends once they have finalized their analysis in the form of an official ISA report. The onus then falls on school leadership to address these issues, and student involvement tends to be less formalized and robust. Second, there is no predefined method for how schools should respond to the ISA report. While this has the advantage of offering flexibility to each medical school, some institutions have taken the approach of simply providing a response summary directly to the LCME (and sometimes to their students) to address the concerns raised in the student-led report. This can undermine the quality improvement process inherent to the self-study period, which the ISA is intended to inform, as there is no direct engagement between the stakeholders who are best suited to foster change: students and their medical school’s leadership. Third, the ISA is only required at the time of re-accreditation, a process that generally takes place every 8 years. As such, it is challenging to know how students feel about institutional changes sparked by the accreditation process, especially if their perception has changed in response to these changes within the timeframe between the ISA survey and the formal LCME site visit.

Though all medical school programs undergo some level of accreditation review on a regular basis, there is a relative dearth of evidence in the literature regarding best practices for engaging students through the LCME process. This paucity was similarly noted by authors from Stony Brook University School of Medicine as they summarized their change management strategy in preparation for their institution’s own LCME site visit in 2011 ( Chandran, Fleit and Shroyer, 2013). Though they provide a helpful overview of student engagement in their institution’s re-accreditation experience, there was no mention regarding if and how students were involved with leading efforts to directly address areas of concern as identified through the ISA. Based on our student experience (KWS, AGB, JCS) in leading the ISA process at our institution, discussion with LCME site visitors, and exchanges with contemporaries at other medical schools, it is our understanding that most schools do not have a formalized process for how they involve students in responding to the ISA data through structural or programmatic changes to the school. To address this gap, we provide a descriptive overview of how students were involved in the re-accreditation in preparation for our institution’s 2019 LCME site visit.

## Methods

### Aims

This study has three key aims to 1) provide a detailed descriptive summary of an innovative model for engaging students throughout the re-accreditation process at Harvard Medical School (HMS), which we refer to as the Independent Student Analysis Action process, or ISA ^2^; 2) summarize levels of key stakeholder engagement, including student body response rates to re-accreditation surveys as well as the number of faculty, staff, and students engaged in ISA ^2^ working groups; and 3) examine stakeholder satisfaction with the ISA ^2^ process and recommendations for further improvement or customization.

### Population Studied and Setting

Findings stem from HMS’ experience with engaging the student body with the re-accreditation process from 2018-2019 in Boston, Massachusetts, in the U.S.

### Study Design

This is a descriptive account of the ISA ^2^ approach based on our collective experiences as student leaders (KWS, AGB, and JCS) and institutional leadership (EMH) in designing and implementing this process. It draws upon our shared expertise and records. All response rates and satisfaction scores come from two anonymous, optional student body surveys conducted in 2018 that were collected as part of a quality improvement effort to incorporate student feedback in the LCME self-study process from 2018-2019 at our institution and included in our institution’s final ISA report.

## Results

### The ISA2 Process

**Figure 1.**
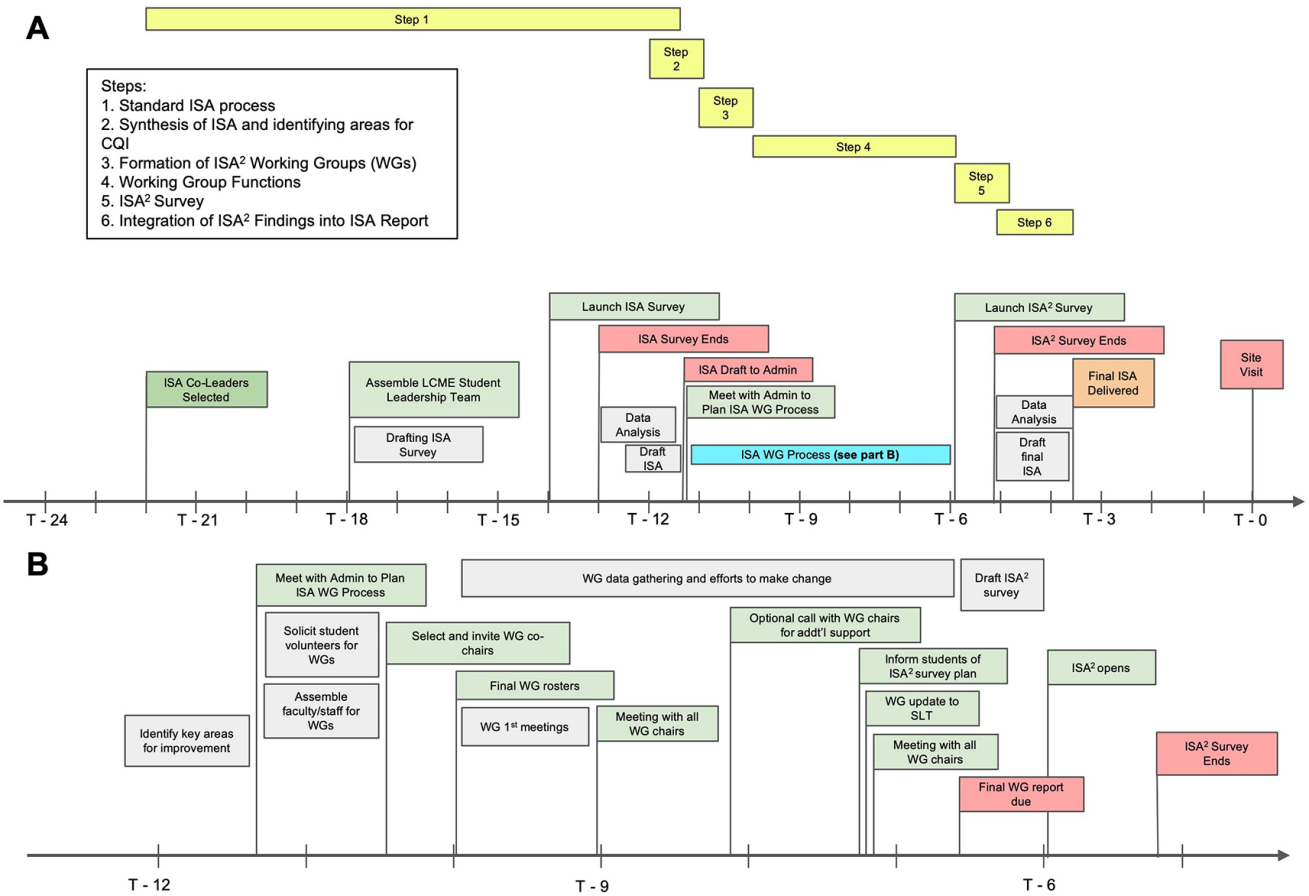
Timeline of Overall ISA Process (Panel A) and the ISA ^2^ process (Panel B). *Figure 1 Notes:* T-0 = Site Visit. A more detailed summary is provided in Table 1. Additional information is available upon request.

The ISA ^2^ process can be summarized in six key steps ( Figure 1; Table 1).

**Table 1.** Key Steps for ISA ^2^ Process (T=0: Site Visit)

ISA ^2^ Key Steps	Months prior to Site Visit (T)	Detail
1. Standard ISA Process	T-22 to T-12	• The LCME provides a detailed guidance document for the ISA, titled: “The Role of Students in the Accreditation of Medical Education programs in the U.S.”. Within this document, the LCME provides a “base” of approximately 70 questions that should be included in every ISA (as these are linked directly into the final Data Collection Instrument (DCI) for the school), but students are welcome to include others. • At our institution, we formed a student leadership team (SLT) to assume the responsibilities as delineated in this document. The SLT was comprised of 19 students across all years, and co-led by 3 students (KWS, AGB, JLS) that represented both curricular tracks. • Some members from the SLT formed a survey design subcommittee to develop, refine, and beta test the survey instrument that would eventually be circulated to the student body. • The final ISA consisted of a 244-question anonymous survey (the required LCME questions, demographic questions, and additional questions that addressed specific aspects of the curriculum and areas of student life at HMS. Nearly all questions were in the requested format of a 4-point satisfaction scale (Very Satisfied, Somewhat Satisfied, Somewhat Dissatisfied, Very Dissatisfied), and included a “Not Applicable” (N/A) option. Free response questions were also included to allow students to elaborate on key strengths of the school and areas that they wish to see improved. • The SLT fielded the ISA 14 months prior to the site visit and had an overall response rate of 98% among all enrolled students. • The SLT led the analysis of the ISA data and summarized its findings in a preliminary ISA report that was delivered to institutional leadership for the self-study process approximately 12 months prior to the LCME site visit.
2. Synthesis of ISA and identifying areas for CQI	T-12 to T-11	• The 3 SLT co-leaders reviewed areas of relatively lower satisfaction as evidenced from ISA analysis with the entire SLT and identified a list of options for priority areas. This was based on both quantitative analysis from satisfaction data as well as qualitative analysis of themes emerging from free responses. Some of this work was concurrent with drafting the preliminary ISA report. • This list generated by the SLT co-leaders was discussed with institutional leadership in order to identify areas that would be most high yield to focus on for CQI process and had synergies with ongoing initiatives through the school’s self-study process. • A total of 11 areas were identified that would benefit from a follow-up survey among the student body to provide the LCME with a more current view of student perceptions to changes implemented during the self-study process: study and relaxation space; career advising; increasing access to diverse faculty and curriculum; wellness and mental health; registrar functions; standardized exam preparation; communication of school policies and updates; building cross-class community; interview day; principal clinical experience (the clerkship year); and pre-clerkship curriculum (1 for each of the two curricular tracks at HMS), these latter two focused on the courses/clerkships with lowest student satisfaction ratings.
3. Formation of ISA ^2^ Working Groups (WGs)	T-11 to T-10	• The SLT co-leaders identified student co-chairs and HMS Deans identified faculty/staff co-chairs who were key leaders/decision-makers already working on the core 11 areas to establish a preliminary co-chair leadership list. • Invitation emails were circulated to student and faculty co-chairs outlining goals and timeline, with nearly 100% acceptance rate. • SLT co-leaders then invited the entire student body to volunteer for a WG. Students could indicate a preference for any WG they wished to join. • All student volunteers were placed on one of their preferred WGs, with each group ranging from 3 to 35 members (note: some of the larger WGs split into sub-WGs). • Created an institutional listserv for all WG members to facilitate communication across all WGs.
4. Working Group (WG) Functions	T-10 to T-7	• WGs had approximately 4 months to meet together and address their charge. The key deliverable was a 1-page summary of “Actions Taken” and “Plans in Place”, which would serve as key content presented to students in the ISA2survey. • SLT co-leaders organized 2 in-person meetings to provide WG leadership with support, allow for direct exchange across WGs, and to gauge progress. The initial meeting was critical for helping to establish connections across the WGs and to ensure progress was being made. These meetings also helped to inform the final format for the content that would be presented to students in the ISA2 survey. • When a final version of the ISA2 survey was created, all WGs were given an opportunity to review the final formatted content, which had been prepared by the 3 SLT co-leaders.
5. ISA ^2^ Survey	T-6	• SLT conducted the follow-up ISA2 survey over a 1-month period via Qualtrics anonymized survey software to the entire student body who was eligible to take the original ISA survey and had not yet graduated. Students were only presented with questions relevant to them (e.g., students in the pre-clinical time were not asked questions related to the clerkship year). • In response to student feedback that they would like to review the content after the completion of the ISA2 survey, we provided all students with a complete summary of all the Actions Taken and Actions Planned across all the WGs on our HMS LCME SLT website for their future reference of resources.
6. Integration of ISA ^2^ Findings into ISA Report	T-4	• SLT co-leaders completed a focused ISA2 report for CQI review to HMS leadership during standing self-study committee meetings. • The SLT ISA writing team integrated all ISA2 findings within the original ISA report. As opposed to providing separate follow-up survey report, we provided an update on how satisfied students were with the changes implemented or planned by the WGs within those core 11 domains, incorporating those updates into the relevant sections and also updating the overall ISA report Executive Summary to provide to the LCME. • We provided our institution with a complete ISA report at T-4, which was part of re-accreditation package submitted with the Data Collection Instrument (DCI) at T-3 to the LCME.

First, we formed a LCME HMS Student Leadership Team (SLT) of 19 students and conducted the typical ISA survey that is expected through the LCME’s published guidance for engaging students in the accreditation process ( Liaison Committee on Medical Education, 2019). The SLT then provided the preliminary ISA report to the entire student body and institutional leadership for the approximately year-long self-study process, which is what we refer to as the “traditional pathway” for most institutions.

Second, and the start of what have been unique steps relative to the traditional pathway, the SLT worked directly with institutional deans to identify key areas of student dissatisfaction emerging from the ISA that were amenable to rapid, high-yield interventions within the institution’s self-study period.

Third, we developed focused, time-limited working groups (WGs), each of which were co-led by 1 student and 1 key relevant faculty/staff member responsible for that domain (e.g., the registrar co-led the registrar functions WG and a clerkship director co-chaired the WG focused on a lower rated clerkship with student representatives). These diverse WGs were charged to review causes for lower satisfaction in their assigned domain, compile existing initiatives designed to address these issues to avoid work duplication, and implement complementary near-term solutions or processes to further improve those areas.

Fourth, the WGs spent approximately four months working on their charges and were empowered to decide how best to address their charge while also being supported by the SLT co-leaders with overarching guidance to harmonize inputs across the WGs. The WGs summarized their findings in a one-page document that included both “actions taken” as well as “plans in place” designed to make improvements within their domain within the remaining academic year ( Table 2).

**Table 2.** Independent Student Analysis Action Process Working Group Deliverables

Section	Detail
Section I: Working Group Information (For Internal Use)	1. LCME ISA working group name (or subgroup name) 2. Working group (or subgroup) co-chairs 3. Working group (or subgroup) members
Section II: Background & Process Information (For Internal Use)	1. Source(s) of Dissatisfaction: *A brief summary of the source(s) of the student dissatisfaction. (Please do not restate the area of dissatisfaction itself based on the ISA results, but rather explain what is underlying this issue).* 2. Working Group Process: *A brief summary of your working group’s process (how often did you meet, did you do any focus groups etc.)*
Section III: Survey Content For ISA2 (length: <1 Page)	1. Actions taken *A bulleted summary of the actions that have already been taken to address student dissatisfaction. This includes actions taken by your working group and actions that have been taken by other groups since the first ISA survey.* 2. Plans in place *A bulleted summary of actions that are definitely being rolled out in Academic Year 2019-these items should already have been designed and are in the process of implementation.*
Section IV: Additional Recommendations (Optional, Not Featured in Survey)	*This includes any recommendations or ideas your working group discussed that you would like to capture for future long-term efforts. These may be relayed to relevant stakeholders (for example, to the Curriculum Committees). This section will NOT be included in the survey.*

Additionally, each group was responsible for presenting their progress to a relevant educational subcommittee - either a pre-existing curricular committee or one formed for the LCME accreditation process - to inform the institution’s ongoing self-study process. This blend of autonomy, support, and accountability empowered each group to work quickly and effectively to optimize their desired output. Further, allowing groups to feature not only actions taken but also the processes in development helped to foster an expectation of CQI beyond the ISA ^2^ and LCME site visit. It also prevented groups from focusing too much on theoretical actions that may be helpful but would not be in place for many years, which can create a false promise for students.

Fifth, we measured the impact of the WGs’ activities through a focused follow-up survey to the student body: the ISA ^2^ survey. Students were presented with the content prepared by each WG (in the form of the aforementioned one-page table) and asked to rate their satisfaction with this area in light of the changes made.

Sixth, we incorporated the ISA ^2^ findings into the final ISA report that was ultimately provided to the LCME. We then analyzed the survey data and incorporated the updated levels of satisfaction into the relevant sections of the initial ISA report. Importantly, the original satisfaction levels were not replaced, but rather a trend in domain satisfaction was established using the ratings from both the ISA and ISA ^2^ surveys.

### Stakeholder Engagement with Re-Accreditation

A total of 821 students completed the initial ISA survey (98%) and 96% (642/666) of eligible student respondents completed the follow-up ISA ^2^ survey at our institution. The WG process engaged over 70 student volunteers along with an additional 40 faculty or staff members.

In terms of working group productivity, the 11 core working groups facilitated 99 actions taken and summarized 83 plans in place, as was captured in the final tables provided to students in the ISA ^2^ survey. For an overall trend, we observed that student satisfaction with each of the domains featured in the ISA ^2^ were higher as compared to levels of satisfaction of those same domains as captured in the ISA. In order to keep ISA participation anonymous, we could not directly measure change in satisfaction among each individual student or how it may have differed by particular demographic groups. Further, between the ISA and ISA ^2^, an entire class of students graduated, thereby narrowing our overall survey eligible respondent list to only those who had completed the ISA and had not yet graduated.

### Stakeholder Satisfaction with the ISA2 Process

**Figure 2.**
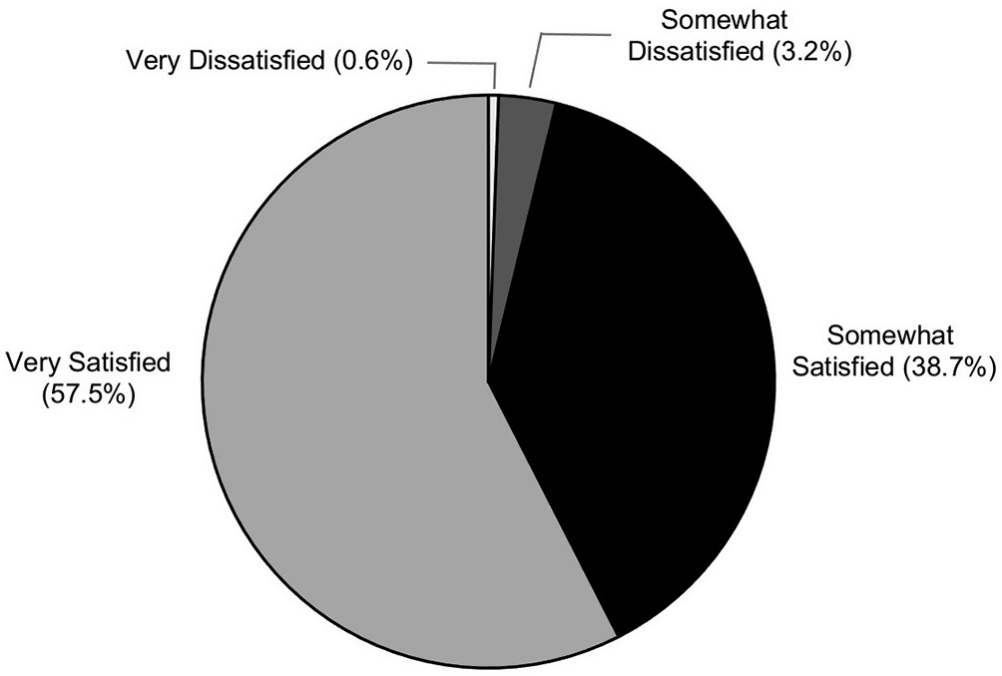
Student body satisfaction with the ISA ^2^ process for utilizing feedback from the ISA survey to address areas of lower satisfaction, n=525 respondents.

Among responding students, 96% (505/525) expressed satisfaction with the ISA ^2^ process as a mechanism for utilizing feedback from the ISA survey to address areas associated with lower student satisfaction ( Figure 2).

Within the optional free response section, student feedback was universally positive and demonstrated appreciation for the work that had been done in response to the ISA survey. Further, deans and faculty involved in the LCME self-study process shared strong, positive sentiments regarding the ISA ^2^ process as a way of focusing improvement efforts during the self-study process. Of note, we have since attempted to incorporate these areas in need of improvement into regular discussions for our Educational Policy and Curriculum Committee, which oversees the medical education at our institution. Having each WG include relevant faculty/staff to that domain as well as ensuring that each WG’s efforts fed into a larger standing body were particularly helpful to broaden the impact of the WG process. Indeed, even after the LCME self-study period concluded, some WGs transformed into ongoing advisory groups to ensure that robust CQI continues.

## Discussion

Taken together, the ISA ^2^ served as a powerful convening mechanism for engaging a large number of students in the CQI process that was integral to our institution’s re-accreditation efforts. Students felt as though their perspectives were being considered at the highest administrative levels during the self-study process and felt that their feedback was influencing near-term solutions that would benefit current and future students. The ISA ^2^ also helped to provide the LCME with a more current, quantitative, and comprehensive trend of student ratings of problematic areas, rather than having the site visitors rely on the viewpoints of the handful of students with whom they met.

Though the ISA ^2^ process was overwhelmingly positive at our institution, our analysis is limited to a single institution and thus may not be generalizable to other settings. Nonetheless, we believe there is potential to customize this model for improvement elsewhere and identify core issues that were helpful to implementing our model. First, it is self-evident but still worth noting that conducting two surveys across the entire student body represents an enormous workload; institutions should ensure that student leaders in their accreditation process are well supported in this endeavor. As student leaders, we appreciated extensive collaboration with the Deans and other faculty, re-accreditation staff, an independent faculty survey design expert, and the Office for Educational Quality Improvement, which helped to optimize the impact of this process. Second, the nature of holding an additional follow-up survey to capture satisfaction following the ISA requires thoughtful planning of the institution’s re-accreditation timeline to ensure there is sufficient time for both endeavors, which means starting the ISA earlier than usual. Third, we did not institute a formalized system to ensure that the “Plans in Place” as listed in the ISA ^2^ survey were actually implemented. Anecdotally, it seems that most of the plans have actually transpired and will be monitored by an existing standing committee; however, a more formal mechanism for supporting these ongoing efforts would be beneficial. This is a current area of focus for remaining members from our SLT to ensure that this follow-up is built into the school’s CQI monitoring system. Related to this, given the standard 8-year gap in accreditation status, it is important for institutions undergoing this process to identify ways to keep students engaged in CQI throughout the interval periods.

## Conclusions

In summary, the ISA ^2^ process helped to engage students in a meaningful way throughout our institution’s LCME re-accreditation self-study period. It brought over 110 students, faculty, and staff together to address pressing issues facing students, allowing all members to co-own the creation of meaningful change. Nearly all students were satisfied with the ISA ^2^, allowing them to voice their perspectives on how to improve the structures influencing their educational experience and that of future students. It is our hope that this innovation can be adapted and further refined by medical schools that strive to partner with their students through the accreditation process and collectively improve their institution.

## Take Home Messages

• The Independent Student Analysis (ISA) is a student-led survey that plays an important role in the Liaison Committee on Medical Education’s (LCME) review of a school’s accreditation status in the United States.
• Here we summarize a novel model for furthering the collaboration between students and faculty beyond the traditional ISA report to implement positive changes throughout the re-accreditation process through what we call the Independent Student Analysis Action (ISA ^2^) model.
• Other medical schools seeking to involve students in their accreditation experience may benefit from customizing this model to their own institution’s needs.

## Notes On Contributors

*Kirstin W. Scott is a fourth-year medical student at Harvard Medical School, Boston, MA. ORCID: https://orcid.org/0000-0002-5415-6479

*Adam G. Berger is a third-year medical student at Harvard Medical School, Boston, MA. ORCID: https://orcid.org/0000-0002-3813-8684

*Jessica C. Stuartis a recent graduate of Harvard Medical School and currently a first-year resident in internal medicine at the Brigham & Women’s Hospital, Boston, MA.

Edward M. Hundert is the Dean for Medical Education and Daniel D. Federman, M.D. Professor in Residence of Global Health and Social Medicine at Harvard Medical School, Boston, MA.

* *These three student authors contributed equally to the development and implementation of this entire ISA ^2^ initiative and the corresponding manuscript enclosed here.*
