# Supplementary material for: Re-engaging students in medical school accreditation: the independent student analysis action (ISA2) approach
Source: MedEdPublish (2016). 2020 Jan 15;9:15. [Version 1] doi: 10.15694/mep.2020.000015.1 (PMC10702661; doi:10.15694/mep.2020.000015.1)
Supplement: Peer review history

# Reviewer report: Reviewer response for version 1

**DOI:** 10.21956/mep.19979.r30386

**Published:** 2020-05-12

**Authors:** Ken Masters

**Affiliations:** 1 Sultan Qaboos University

This review has been migrated. The reviewer awarded 4 stars out of 5 An interesting paper on re-engaging students in medical school (re-)accreditation. The paper clearly lays out the problem of the need to engage students properly in the evaluation process for accreditation, and then lays out in some detail, the process followed at Harvard Medical School. The paper’s value lies in the details of the approach taken. While many of these will be specific to the situation only, most have enough information for principles to be derived and for corresponding issues to be addressed and actions to be taken.In addition, a reader would hopefully take the approach that the purpose is not to “get re-accredited”, but rather to address students’ concerns; accreditation is merely a confirmation of a job well done. I would have felt a little more comfortable if that point had been explicitly and strongly made in the paper, otherwise, there is a risk that the process becomes an aim at a standardised tick-box exercise.A minor quibble: Figure 2 really does not serve much purpose: the information would be better displayed as a table, with raw numbers as well as percentages).There are a few other minor issues, as noted by the first reviewer, so there is no need for me to raise them again.

Reviewer Expertise:

NA

No decision status is available

# Reviewer report: Reviewer response for version 1

**DOI:** 10.21956/mep.19979.r30385

**Published:** 2020-01-23

**Authors:** P Ravi Shankar

**Affiliations:** 1 American International Medical University

This review has been migrated. The reviewer awarded 4 stars out of 5 I enjoyed reading this well-written paper. Students have co-authored the paper with a faculty member. I have been involved with accreditation in the Caribbean by CAAM-HP and ACCM who follow a similar process to the LCME. The Independent Student Analysis (ISA) is an important part of the self-study. There is also a gap of nearly a year between submission of the self-study and the site visit. The authors describe a process of taking action on the deficiencies noted during the ISA in a time bound manner. I enjoyed reading through their description and believe that with a few modifications it can be applied to schools in other locations. Doing two large student surveys within a period of less than a year can be challenging as mentioned by the authors. Figure 1 is a bit difficult to read. I agree with the other reviewer that the LCME (and the CAAM-HP) accredit a program and a not a school. I am sure all medical educators will find this manuscript to be of interest.

Reviewer Expertise:

NA

No decision status is available

# Reviewer report: Reviewer response for version 1

**DOI:** 10.21956/mep.19979.r30387

**Published:** 2020-01-15

**Authors:** Robert Hash

**Affiliations:** 1 American Medical Association

This review has been migrated. The reviewer awarded 5 stars out of 5 This is a well-written description of how the school uses the ISA to inform the accreditation process, and more importantly, to improve the quality of the educational program and student experience. The later are goals of the self-study. The article emphasizes that the ISA process and results can be used well beyond the accreditation process to engage learners, faculty, and administration in collaborative activities to improve the educational program, learning experience, and student support. A few minor inaccuracies need to be addressed. These inaccuracies in no way diminish the importance of the work described in this article. The LCME accredits medical education programs, not medical schools. The difference is subtle but important. Also, the LCME survey teams makes recommendations regarding the 95 Elements that comprise the 12 accreditation Standards, but not the Standards themselves. The LCME can agree with or change those recommendations, based on review of the team report and accompanying data, which includes the ISA. Determining compliance with the Standards is the sole responsibility of the LCME. This article serves as a roadmap for programs and institutions (not just medical education) to use student engagement in accreditation to improve program quality beyond episodic accreditation. Well done and thank you for the work.

Reviewer Expertise:

NA

No decision status is available
